# Retinal capillary perfusion heterogeneity in diabetic retinopathy detected by optical coherence tomography angiography

**DOI:** 10.1186/s40942-024-00528-6

**Published:** 2024-01-25

**Authors:** Po Hsiang (Shawn) Yuan, Arman Athwal, Mena Shalaby, Andrew Mehnert, Dao-Yi Yu, Rony C. Preti, Marinko Sarunic, Eduardo V. Navajas

**Affiliations:** 1 Department of Ophthalmology and Visual Sciences, Faculty of Medicine, University of British Columbia, Vancouver, BC Canada; 2 School of Engineering Science, Simon Fraser University, Burnaby, BC Canada; 3 Centre for Ophthalmology and Visual Science, University of Western Australia, Perth, Australia; 4 Lions Eye Institute, Nedlands, WA Australia; 5 Department of Ophthalmology, University of Sao Paulo, Sau Paulo, Brazil; 6 Institute of Ophthalmology, University College London, London, UK; 7 Department of Medical Physics and Biomedical Engineering, University College London, London, UK; 8 Eye Care Centre at Vancouver General Hospital, 2550 Willow Street, Vancouver, BC V5Z 0A6 Canada

**Keywords:** Diabetic retinopathy, Optical coherence tomography angiography (OCTA), Perfusion heterogeneity, Retinal imaging, Retinal perfusion, Segmentation

## Abstract

**Background:**

Diabetic retinopathy (DR) is a leading cause of blindness and involves retinal capillary damage, microaneurysms, and altered blood flow regulation. Optical coherence tomography angiography (OCTA) is a non-invasive way of visualizing retinal vasculature but has not been used extensively to study blood flow heterogeneity. The purpose of this study is to detect and quantify blood flow heterogeneity utilizing en-face swept source OCTA in patients with DR.

**Methods:**

This is a prospective clinical study which examined patients with either type 1 or 2 diabetes mellitus. Each included eye was graded clinically as no DR, mild DR, or moderate-severe DR. Ten consecutive *en face* 6 × 6 mm foveal SS-OCTA images were obtained from each eye using a PLEX Elite 9000 (Zeiss Meditec, Dublin, CA). Built-in fixation-tracking, follow-up functions were utilized to reduce motion artifacts and ensure same location imaging in sequential frames. Images of the superficial and deep vascular complexes (SVC and DVC) were arranged in temporal stacks of 10 and registered to a reference frame for segmentation using a deep neural network. The vessel segmentation was then masked onto each stack to calculate the pixel intensity coefficient of variance (PICoV) and map the spatiotemporal perfusion heterogeneity of each stack.

**Results:**

Twenty-nine eyes were included: 7 controls, 7 diabetics with no DR, 8 mild DR, and 7 moderate-severe DR. The PICoV correlated significantly and positively with DR severity. In patients with DR, the perfusion heterogeneity was higher in the temporal half of the macula, particularly in areas of capillary dropout. PICoV also correlates as expected with the established OCTA metrics of perfusion density and vessel density.

**Conclusion:**

PICoV is a novel way to analyze OCTA imaging and quantify perfusion heterogeneity. Retinal capillary perfusion heterogeneity in both the SVC and DVC increased with DR severity. This may be related to the loss of retinal capillary perfusion autoregulation in diabetic retinopathy.

## Background

Diabetic retinopathy (DR) affects about one third of patients with diabetes, estimated at 145 million people worldwide, and is the leading cause of new legal blindness in the working age population [1, 2]. The pathogenesis of DR includes the loss of pericytes and endothelial cells at the capillary level, leading to microaneurysm formation, capillary occlusion, and retinal hypoxia which results in angiogenic processes such as the upregulation of vascular endothelial growth factor (VEGF) [2, 3]. With the progression of DR, further signs of vaso-occlusion may develop such as cotton wool spots, venous beading, intraretinal microvascular abnormalities, and increased amounts of intraretinal hemorrhage, which are features seen in advanced non-proliferative DR [3–5].

The intermittent blood flow through capillaries, turning on and off every few seconds or minutes, is a phenomenon called vasomotion and is caused by the periodical contractions of terminal arterioles and pre-capillary sphincters [6]. This perfusion heterogeneity in retinal capillaries is expected in healthy individuals and is thought to be one of the most important features to determine the autoregulatory capacity of capillaries to fluctuating local oxygen and metabolic demands [6, 7]. In addition to blood flow regulation proportional to local metabolic demands, the retinal autoregulation control of capillary flow responds to fluctuations in systemic arterial pressure [6, 7]. This mechanism helps prevent endothelial cell damage and subsequent leakage of plasma or lipids during sudden increases in systemic arterial pressure by reducing transluminal capillary pressure gradient [7]. Diabetes mellitus (DM) induced disruption of the retinal autoregulation of capillary flow may result in the increased risk of capillary wall damage due to the lack of protection against increased systemic arterial pressure [6, 8].

Optical coherence tomography angiography (OCTA) is a depth-resolved, non-invasive, and dye-free imaging modality that uses flow-based contrast to visualize the multi-layered retinal circulation [3, 5, 7]. OCTA allows quantitative measurements to describe the status of the retinal vasculature. Previous studies have shown that vessel and perfusion densities (VD and PD, respectively), quantitative metrics generated by most commercial OCTAs, have an intra-visit variation ranging from 2.1% to 6.8% [9–13]. However, there are a lack of studies using quantitative OCTA vascular metrics to assess for pixel-level retinal perfusion heterogeneity. The purpose of this study was to serially acquire *en-face* OCTA images to calculate the intra-visit pixel intensity coefficient of variation (PICoV) in normal and diabetic patients. We hypothesize that the PICoV can be used as measurement of retinal spatial and temporal blood flow heterogeneity.

## Methods

### Study patients

This prospective, cross-sectional clinical trial was approved by the institutional review board of the University of British Columbia (UBC), Vancouver, British Columbia, Canada (H18-02095), registered at clinicaltrials.gov (NCT03765112) and adhered to the tenants of The Declaration of Helsinki [14]. Patients were recruited at the Department of Ophthalmology and Visual Sciences (UBC, Vancouver, BC, Canada) and written informed consent was obtained. Patients with Type 1 or 2 DM, DR of each severity level, and healthy controls that had 10 serially acquired *en-face* OCTA images were included. DR severity levels were determined clinically by an experienced retinal ophthalmologist (EVN) following ETDRS classifications [15]. The following exclusion criteria were applied: media opacities, active intraocular inflammation, retinal disease other than DR including signs of age-related macular degeneration, drusen, retinal artery or vein occlusion, structural damage to the macula, history of vitrectomy, glaucoma or use of intraocular pressure lowering eye drops, history of glaucoma surgery, intraocular surgery (including cataract surgery, YAG laser, panretinal laser photocoagulation, intravitreal injection of an anti-VEGF agent or corticosteroids) within 3 months prior to image acquisition. Medical history was obtained including type of diabetes, duration of diabetes (defined by the start of anti-diabetic medication), latest A1c level, height and weight, history of high blood pressure or ongoing anti-hypertensive medication use, history of cardiovascular events (including angina pectoris, myocardial infarction, bypass surgery, stent implantation, ischemic cardiac disease, stroke), smoking habits, history of ocular surgery including lasers and intravitreal injection therapies.

### Optical coherence tomography angiography imaging protocol

All patients were imaged with the commercially available PLEX Elite 9000© OCTA (Zeiss Meditec, Dublin, CA), using a 6 × 6-mm scanning protocol while sampling at a 500 × 500 resolution at a rate of 100,000 A-scans per second. The A-scan depth was 3 mm, the axial resolution 6.3 μm, and the transverse resolution 20 μm, as listed in the product specifications. Images were acquired using the built-in fixation-tracking function which detects and corrects for micro saccadic eye movement and blinking. Ten consecutive 6 × 6 mm *en-face* images were serially acquired using the built-in registration function. *En-face* images of the superficial vascular complex and deep vascular complex (SVC and DVC) were obtained through automatic layer segmentation by the device. The SVC included the radial peripapillary capillary and superficial capillary plexus located between the inner limiting membrane and the posterior boundary of the inner plexiform layer (IPL). The DVC included the intermediate capillary plexus and deep capillary plexus from the posterior boundary of the IPL to the posterior bound of the outer plexiform layer.

To reduce the influence of potential confounders, a strict image quality criterion was implemented where images labeled with less than 8/10 signal strength (as evaluated by the proprietary Zeiss software) were not saved. The majority of images analyzed held a signal strength score of 10/10. Images were manually inspected during acquisition for signs of improper focus and other artifacts like floaters. If such artifacts were present, further images were acquired to ensure at least ten ‘clean’ images were acquired per eye. Finally, a manual review of the full data set revealed no significant imaging artifacts that could confound the PICoV perfusion heterogeneity signal.

### Image registration and averaging

The ten sequentially acquired SVC and DVC images of each eye were downloaded from the acquisition device and imported into MATLAB (2019b; MathWorks) for image processing. Each set of 10 images was arranged into three-dimensional image stacks for temporal variation analysis. Although OCTA images are aligned by the PLEX Elite system during acquisition, minor residual motion artifact remains in the output images. This was corrected via a two-step registration process. A preliminary pre-processing step involves the manual selection of a ‘template’ image to which the remaining images in the temporal stack are registered. The image containing the least amount of motion artifact and highest overall signal quality was chosen as the template. The first registration step was a cross-correlation-based registration in which images are shifted laterally in the fast- and slow-scan directions. The second registration step is a more precise non-rigid registration, utilizing a MATLAB-based implementation of Thirion's demons algorithm in the form of a non-parametric diffeomorphic image registration algorithm [16]. Finally, the stack of registered images was averaged to create an image with high signal-to-noise and contrast-to-noise ratios for use as input to the neural-network-based vessel segmentation algorithm. Outputs from this framework result in high quality OCTA images with clearly traced retinal vessels [17]. Projection artifacts of superficial vessels into DVC images were attenuated for purposes of PICoV, PD, and VD calculations by segmentation and subtraction of the largest SVC vessel projections in the DVC images.

### Vessel segmentation via deep neural network

Following registration of the 10 sequentially acquired en face images, the vascular signal was isolated from background noise via automatic vessel segmentation by a deep neural network (DNN). This machine-learning approach to retinal vessel segmentation was shown to outperform previous segmentation methods such as intensity thresholding and morphological filters [18]. The training set for this neural network model incorporated control and DR patient OCTA 3 × 3 mm images from a Zeiss PLEX Elite 9000 device. The neural network takes as input an averaged grayscale OCTA image and outputs a ‘probability map’ of the same size, where each pixel is assigned a value between 0 and 1, describing the confidence in each pixel corresponding to a blood vessel. The probability maps were thresholded at a value of 0.5, used as a mask, and multiplied with the registered image stack to reduce background noise.

### Pixel intensity coefficient of variation calculation

**Fig. 1:**
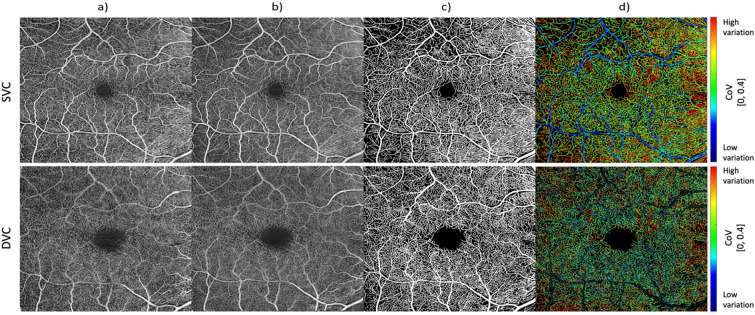
Processing pipeline of OCTA *en-face* images of the superior vascular complex (top row) and deep vascular plexus (bottom row) of a right eye (OD) with mild diabetic retinopathy. **a** Selected template 6 × 6 mm foveal centered image for registration reference **b** Averaged image after registration of 10 serially acquired images. **c** Deep Neural Network (DNN) vessel segmented image. **d** Pixel Intensity Coefficient of Variation colour map. Colour map values range from blue (no variation) to red (high variation), and were upper-end capped at 0.4 and subsequently remapped between 0 and 1 to yield a higher dynamic range

**Fig. 2:**
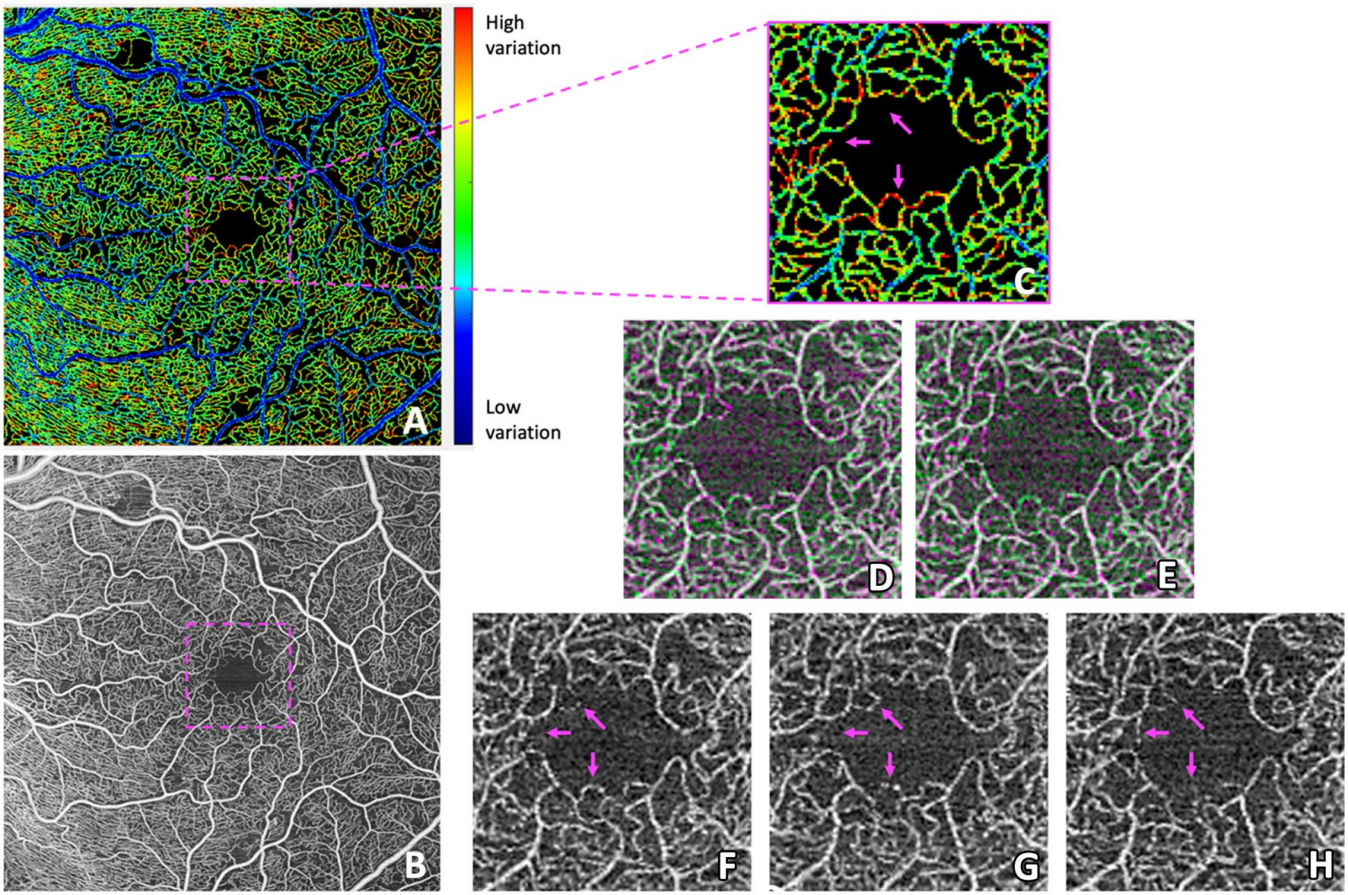
**A** The 6 × 6 mm foveal centered Pixel Intensity Coefficient of Variation (PICoV) image of the superficial vascular complex for a right eye (OD) with mild diabetic retinopathy. **B** The 6 × 6 mm averaged OCTA image created from the same registered stack as panel A. **C** Magnification of panel A showing high temporal filling heterogeneity in the inner foveal avascular zone (FAZ) ring, while the surrounding capillary bed remains mostly static. **D**–**E** Registered image overlays of non-template SVC images, demonstrating registration efficacy. White overlay pixels denote common pixels shared between two images, while green and magenta overlay pixels denote pixels that differ in intensity between two images. Panel **D** is an overlay of panels **F** and **G**, while panel **E** is an overlay of panels **F** and **H**. **F**–**H** Individual OCTA images magnified to the FAZ region, illustrating how substantial intensity variation of individual capillaries manifest as high (red) PICoV signal in panels **A** and **C**. Magenta arrows indicate capillary segments featuring particularly high temporal variation relative to their surrounding vessels. Note how some FAZ capillaries are visible in one frame, while entirely absent in another

**Fig. 3:**
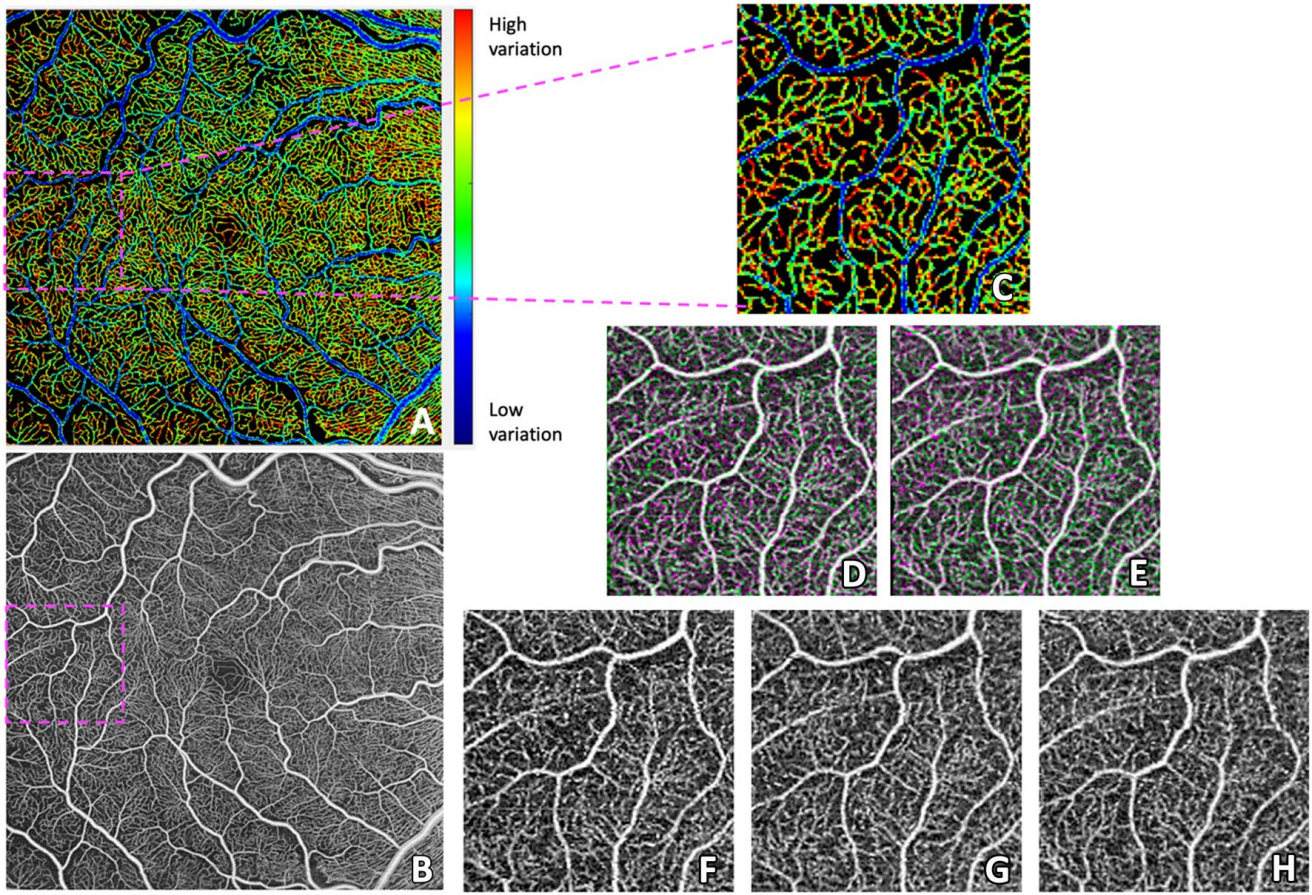
**A** The 6 × 6 mm foveal centered Pixel Intensity Coefficient of Variation (PICoV) image of the superficial vascular complex for a right eye (OD) with mild diabetic retinopathy. **B** The 6 × 6 mm averaged OCTA image created from the same registered stack as panel **A**. **C** Magnification of panel **A** showing high temporal filling heterogeneity in certain capillaries within the temporal retina. **D**–**E** Registered image overlays of non-template SVC images, demonstrating registration efficacy. White overlay pixels denote common pixels shared between two images, while green and magenta overlay pixels denote pixels that differ in intensity between two images. Panel **D** is an overlay of panels **F** and **G**, while panel **E** is an overlay of panels **F** and **H**. **F**–**H** Individual OCTA images magnified to the temporal region, illustrating how substantial intensity variation of individual capillaries manifest as high (red) PICoV signal in panels **A** and **C**

The final processing step to quantify the retinal capillary perfusion heterogeneity is the PICoV calculation. PICoV is defined as the ratio of the standard deviation to the mean of a corresponding input vector and measures the pixel-wise variability of vascular signal between the serially acquired OCTA images [7]. The input vector into each PICoV calculation is an array containing the 10 intensity values of a pixel at identical locations of the OCTA image, for all 10 acquired and registered en-face images. The calculated PICoV values were then displayed by mapping the values to a false colour map. Figure 1 gives a closer look at the resulting PICoV image from the registration, averaging, and DNN segmentation pipeline. Meanwhile, Fig. 2 illustrates how single-image capillary variations manifest as high PICoV values and is a good example of how capillary shunting with a frequency on the order of 10 s of seconds is particularly visible in the inner-ring capillaries of the FAZ. Figure 3 offers a similar example, where rapid capillary shunting is observable in the temporal capillaries. Both figures demonstrate how careful registration of serially acquired images facilitate this heterogeneity analysis. When interpreting these colorized PICoV images, it should be noted that the largest SVC vessels saturate the OCTA signal irrespective of potential flow variations, so the PICoV value assigned to these vessels (which are predominantly minimal/blue in our Figures) should effectively be considered as unknown [19].

### Perfusion density and vessel density calculation

In an effort to tie the novel PICoV metric to existing, well-established metrics, PD and VD were also calculated. Using the DNN-based vessel segmentation masks described previously, PD was defined as the ratio of the total number of ‘1’ pixels to ‘0’ pixels in a segmented and binarized OCTA image. Meanwhile, VD was defined as the same ratio but derived from the skeletonized version of PD (i.e., the PD metric is influenced by vessel area, whereas VD is influenced only by the number of skeletonized vessel segments). With these definitions in mind, the PD and VD metrics were calculated for every single raw OCTA image in each set of 10 images per eye, and the mean and standard deviations of these sets of 10 PD & VD values were calculated. It was hypothesized that increasing DR severity and decreased PD/VD (a well-established correlation) correlate with increased capillary perfusion heterogeneity (i.e., higher PICoV) while increased standard deviations of PD/VD correlate directly with higher PICoV. These degrees of correlation were quantified by the Pearson correlation coefficient. P-values were then calculated from each Pearson correlation coefficient to determine whether PICoV was significantly correlated to any of the PD or VD metrics.

### Statistical analysis

Kruskal–Wallis tests were applied to both the SVC and DVC data to determine whether or not there was any significance between the DR severity groups. Given the significant Kruskal–Wallis tests in both retinal vascular layers, post-hoc Nemenyi tests were conducted to evaluate between which DR severity groups the significance was detected and account for Type 1 errors introduced by multiple comparisons. Post-hoc Nemenyi tests were chosen due to limited sample sizes and PICoV values being bound as > 0, both contributing to the rejection of a Gaussian distribution assumption. If the p-value is less than 5% or the confidence interval crosses 1, the null hypothesis is rejected and indicates a significant PICoV difference between two groups.

Each stack of 6 × 6 mm OCTA images were then divided into equal 3 × 3 mm quadrants (Superior Temporal—ST, Superior Nasal—SN, Inferior Temporal—IT, Inferior Nasal—IN) and PICoV values were obtained for each quadrant, in each retinal layer. Kruskal–Wallis tests were then similarly applied to determine whether or not significant inter-quadrant differences existed. The significant Kruskal–Wallis tests prompted post-hoc Tukey tests to determine which specific inter-quadrant differences were significant. This DR-severity-sparing comparison is valid because each eye contributes equally to each quadrant. DR severity-stratified inter-quadrant analyses were not conducted due to limited sample sizes and an exponential increase in the likelihood of Type 1 error resulting from the repeated comparisons.

## Results

The patient demographics and diabetes information are shown in Table 1. Out of the 22 study participants, there were 5–6 participants in each study group: healthy controls, no DR, mild DR, and moderate-severe DR. The number of eyes in each group were 7, 7, 8, and 7 respectively. There were no significant differences in age, sex, BMI, and A1c between each of the study groups, although duration of diabetes was correlated with severity of DR. There were 3, 2, and 3 hypertensive patients in the no DR, mild DR, and moderate-severe DR groups respectively. All patients had blood pressures well controlled (< 140 mmHg) with medications at the time of study enrollment.

**Table 1:** Demographics of study participants.

	Healthy controls (n = 5)	No DR (n = 5)	Mild DR (n = 5)	Mod-severe DR (n = 5)
Number of eyes	7	7	8	7
Age (range)	53.8 (26–78)	65.2 (56–73)	61.6 (56–69)	62.3 (52–68)
Sex (M:F)	2:3	2:3	4:1	2:3
Diabetes duration in years (range)	N/A	9.4 (5–19)	14.3 (1–50)	18.7 (10–32)
Hemoglobin A1c (range)	N/A	7.8 (5.1–13)	6.6 (6.0–7.2)	7.1 (6.1–7.8)
Insulin use	N/A	1	1	1

*DR* diabetic retinopathy

**Fig. 4:**
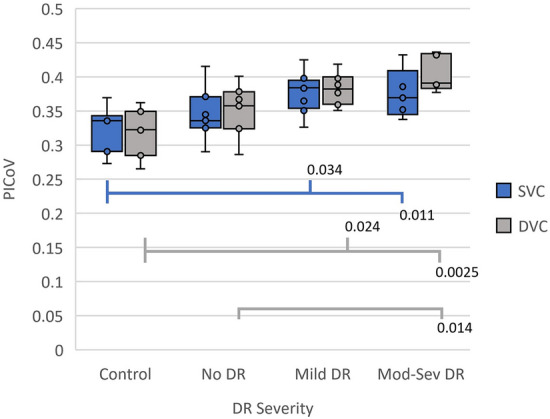
Distribution of the Pixel Intensity Coefficient of Variances (PICoV) in both the superficial vascular complex (SVC) and deep vascular complex (DVC), stratified by diabetic retinopathy (DR) severity. Post-hoc Nemenyi tests were conducted after significant Kruskal–Wallis tests to compare the PICoV values between each DR group within both vascular complexes. P-values of significant differences determined via post-hoc Nemenyi tests are shown; a p-value of < 0.05 indicates significance between the two groups with Type 1 error inflation correction included by the post-hoc Nemenyi test. Each circle represents a single PICoV value associated with 10 serial optical coherence tomography angiography images

**Fig. 5:**
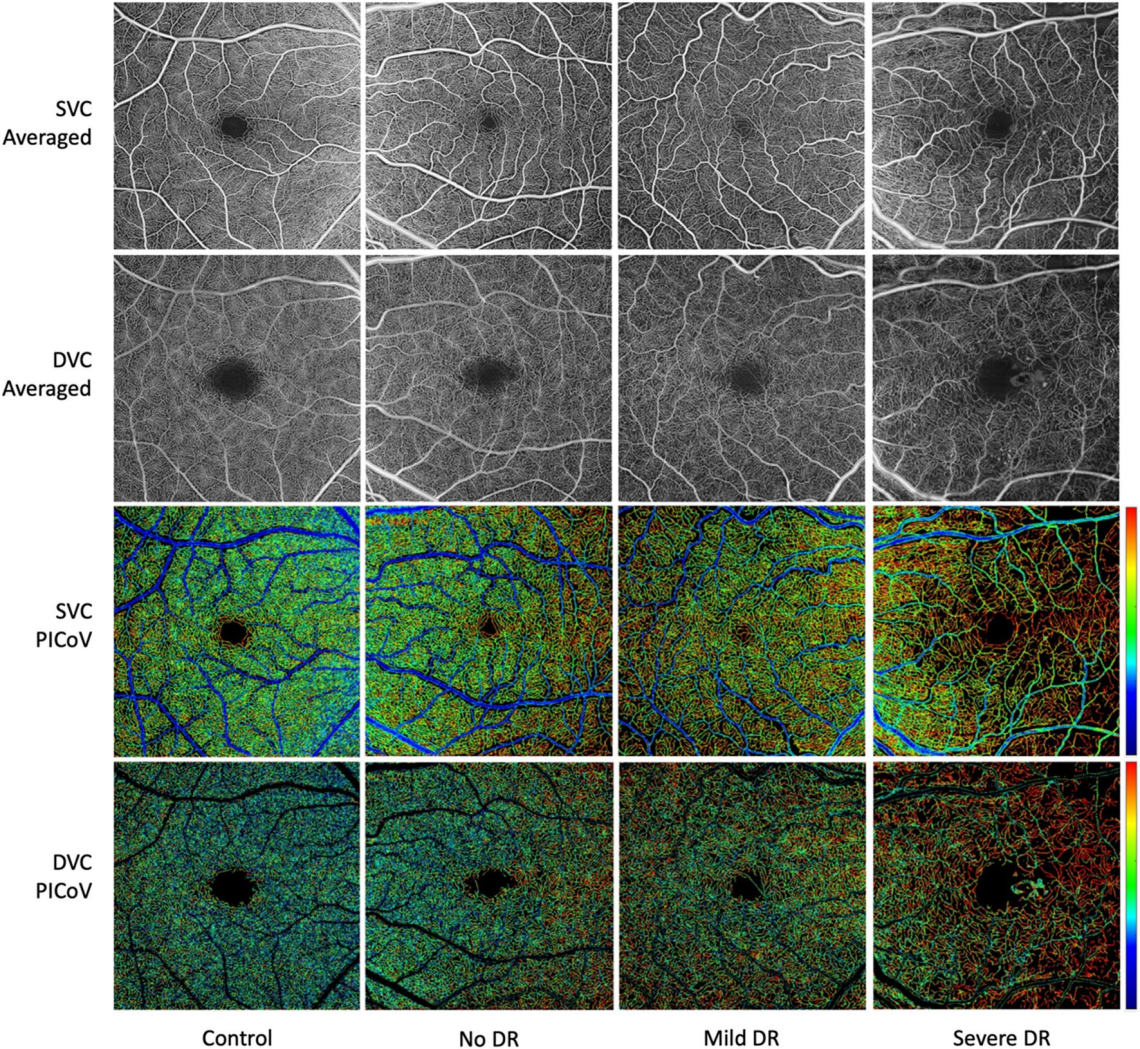
Representative comparison between four eyes within different DR severity classifications, where each column is an eye with different DR classification. First row: averaged OCTA images of the superficial vascular complex (SVC). Second row: averaged OCTA images of the deep vascular complex (DVC). Third row: pixel intensity coefficient of variance (PICoV) maps of the SVC. Fourth row: PICoV maps of the DVC. High-quality averaged images are produced via image registration of 10 serially acquired 6 × 6 mm foveal-centered OCTA images and pixel-wise averaging. PICoV maps are generated by masking a Deep Neural Network vessel segmentation over the registered image stack to reveal areas of perfusion heterogeneity from blue (no variation) to red (most variation)

**Fig. 6:**
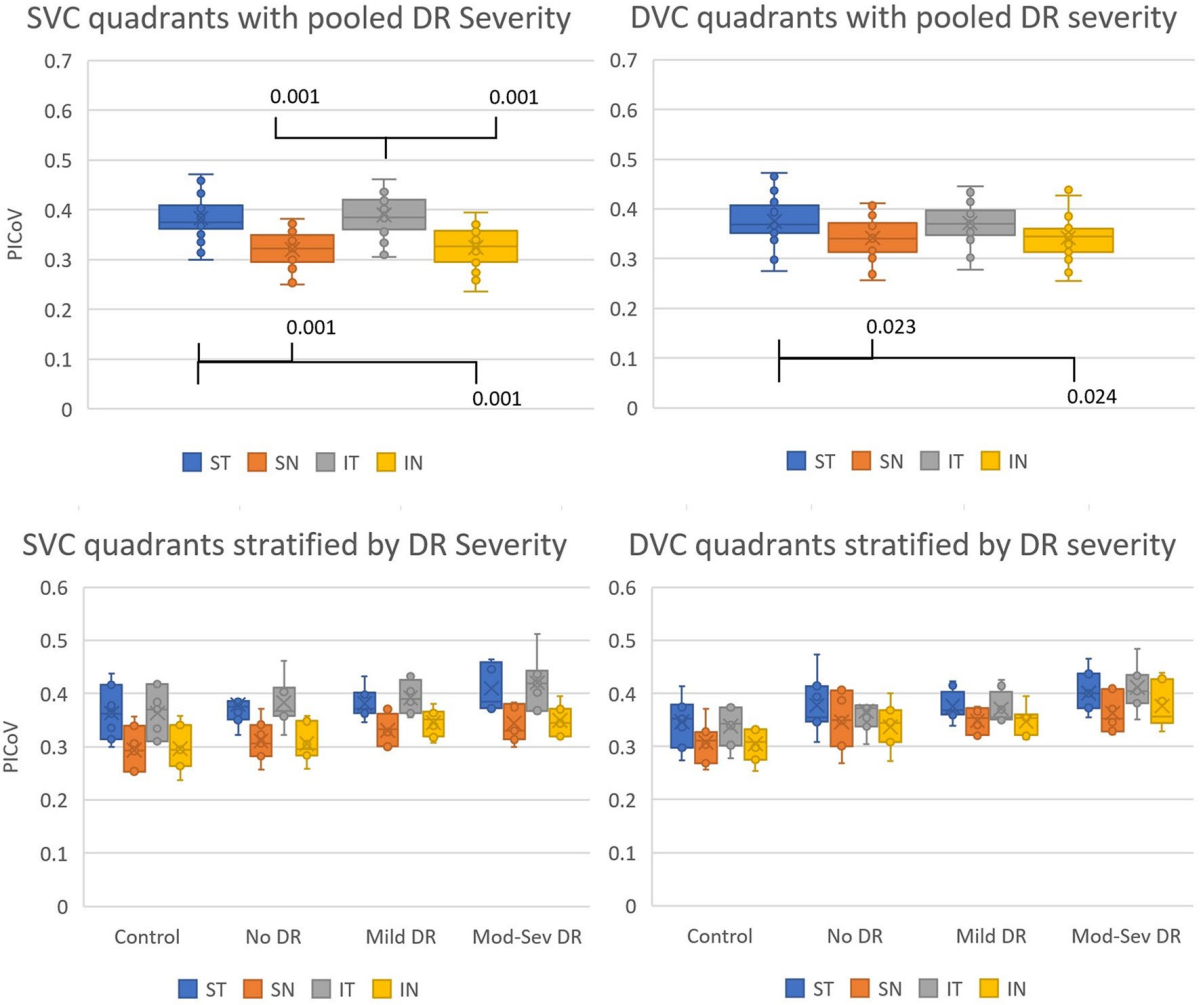
Comparison of Pixel Intensity Coefficient of Variance (PICoV) distributions in each quadrant (ST = Superior Temporal; SN = Superior Nasal; IT = Inferior Temporal; IN = Inferior Nasal) of both the superficial vascular complex (SVC) and the deep vascular complex (DVC). Quadrants are divided equally from 6 × 6 mm fovea-centered OCTA images. Significant Kruskal–Wallis tests provided evidence that there were inter-quadrant PICoV differences in a pooled analysis of all DR groups. Post-hoc Tukey tests with correction of Type 1 error inflation from repeated comparisons were conducted and the resulting p-values of significant relationships are shown. Each circle represents a PICoV value unique in quadrant and eye. Means of each group are shown by X

A positive correlation between PICoV and DR severity in both the SVC and DVC is shown in Fig. 4. There were significant differences (p < 0.05) in PICoV between healthy controls and the groups with DR (mild DR and moderate-severe DR), in both the SVC and DVC. Furthermore, a significant difference between the no DR diabetic group and the moderate-severe DR was observed in the DVC layer. The post-hoc Nemenyi-adjusted p-values of these significant PICoV differences between DR groups are also reported in Fig. 4. Sample PICoV maps from each DR severity group and each vascular layer are shown in Fig. 5 for a visual representation of this relationship. The inter-quadrant analysis of PICoV values indicated that in a 6 × 6 mm *en face* OCTA image, both the ST and IT quadrants have significantly higher PICoV values than either of the nasal quadrants in the SVC (Fig. 6). However, in the DVC, only the ST quadrant had significantly a higher PICoV than the 2 nasal quadrants.

A summary of the relationships between the VD/PD metrics and DR severity are reported in Table 2. The relationships between PICoV and VD/PD are also reported via the Pearson’s correlation coefficient “r”, indicating the direction (positive or negative) and strength of the correlation. Using post-hoc Tukey tests to correct for multiple comparisons, we found significant differences (p < 0.01) within each row when comparing groups with No DR (control and no DR) against groups with DR (mild and moderate-severe DR). In both the SVC and DVC layers, significant negative correlations between PICoV-Mean VD and PICoV-Mean PD were observed. When examining the correlation between PICoV and the standard deviations of PD and VD, a statistically significant positive correlation was observed only in the DVC layer and was weaker than the Mean VD and PD relationships, and for conciseness is not reported.

**Table 2:** Summary of the mean vessel densities (VD) and perfusion densities (PD) in each diabetic retinopathy (DR) severity group, in both vascular complexes

	Metric (r)	Control	No DR	Mild DR	Moderate-Severe DR
SVC	Mean VD (− 0.6284)	0.1288	0.1221	0.1108	0.1028
	Mean PD (− 0.6709)	0.5748	0.5474	0.5043	0.5031
DVC	Mean VD (− 0.8199)	0.1252	0.1169	0.1018	0.0953
	Mean PD (− 0.7803)	0.5945	0.5614	0.4932	0.4854

Using post-hoc Tukey tests to correct for multiple comparisons, we found significant differences (p < 0.01) within each row when comparing groups with No DR (control and no DR) against groups with DR (mild and moderate-severe DR). Pearson's correlation coefficient (r) between each metric and Pixel Intensity Coefficient of Variance is reported in brackets

SVIC Superior vascular complex, *DVC* Deep vascular complex

## Discussion

The results of this study provide evidence that the PICoV of the SVC and DVC increases with DR severity. This increased perfusion heterogeneity variation with advancing DR suggests a disruption of vasomotion, the regulatory mechanism that control intermittent blood flow in the retinal capillaries to local metabolic demands [6–8]. The most important factor affecting vasomotion is oxygen concentration in the tissues [6, 7]. It is possible that in DR-induced retinal hypoxia, the intermittent periods of capillary blood flow occur more often which may translate to an increase in PICoV. In addition, it is possible that DM-induced loss of retinal capillary flow autoregulation to systemic arterial pressure would allow the fluctuations in systemic perfusion pressure to be increasingly transferred to the retinal capillaries, contributing to a higher PICoV. As mean arterial pressures were not obtained from our study patients, this is a potential confounding variable which limits our study. However, no patients had uncontrolled hypertension at the time of study enrollment and image acquisition.

Another notable finding in this study is the distribution of spatial perfusion heterogeneity in the retinal capillaries. For both the SVC and DVC, the PICoV was higher in the temporal half (IT and ST quadrants) of the 6 × 6 mm *en-face* OCTA images. This distribution is correlated with the spatial heterogeneity of the oxygen tension in the retina. Indeed, Yu et al. showed that the retina supplied by the DVC has a low oxygen tension being close to critical levels for neuron survival even in normal retinas [7]. The temporal half of the macula has a lower vascular density than the nasal half and is more prone to the development of microvascular changes in DR [14, 20–22]. A study examining the spatial bias of capillary loss in 6 × 6 mm OCTA images of DR patients showed that capillary dropouts were most frequently observed in the temporal macula [20]. Because tissue oxygen concentration affects vasomotion and the frequency of opening and closure of the capillary networks, areas of the retina with lower oxygen tension such as the temporal half of the macula may exhibit a higher blood flow variation, reflected here by higher PICoV values [7, 20, 21].

A review of OCTA literature identified a trend towards larger foveal avascular zones (FAZ) in patients with diabetes due to foveal capillary non-perfusion [22]. Although our PICoV analysis did not directly examine the FAZ, there was indeed interframe heterogeneity suggesting increased vasomotion in the capillaries surrounding the FAZ (Fig. 2). We suspect that this vasomotion, which is affected by tissue oxygen concentration as previously described, may be an earlier presentation of the permanent capillary loss resulting in enlarged FAZs, reported by previous studies examining microvascular changes in DR [23, 24]. Previous studies have also showed negative relationships between VD and PD and DR severity, which are replicated in our study [11, 21, 25]. In addition, we have shown the relationship of PICoV with these established metrics, and the results were significantly and negatively correlated, as hypothesized.

We acknowledge the limitations of this study. First, while a correlation was found between PICoV and DR severity, the sample size of 29 eyes is too small to draw statistically significant conclusions about the broader population affected by DR. Second, the PICoV analysis pipeline assumes that all serially acquired OCTA volumes are layer-wise segmented and processed consistently. It is possible that some perfusion heterogeneity identified by our analysis may be attributed to variation in the acquisition machine’s image processing and layer segmentation algorithm. Third, the acquisition device’s hardware-based motion tracking system necessarily induces a non-constant acquisition time between volumes. The consequence is that the temporal information of image pixels is lost (i.e., the time interval between pixels within the same image is unknown). Considering this non-constant acquisition time, as well as the non-constant inter-image acquisition time between the 10 serially acquired images, an important implication arises: the pulsatile nature of retinal blood flow and its potential impact on the measured PICoV signal cannot be accounted for [26]. To investigate this effect, ideally the OCTA signal should be measured at a sub-second temporal spacing between volumes, but the necessary hardware for performing said investigation is out of the scope of this study. Finally, it should be noted that since the PICoV values were calculated on a pixel-by-pixel basis, larger vessels have a greater influence on the PICoV value of each image. This is demonstrated in the SVC, where large vessels account for more pixels. As these large SVC vessels saturate the OCTA signal, this may help explain the stronger correlations found in the DVC layer, in which we removed such vessels altogether to account for projection artifacts. Future studies are necessary to determine the role of DR in affecting the retinal perfusion heterogeneity.

## Conclusions

This study introduces PICoV as a novel approach towards analyzing OCTA imaging which relies on serial imaging, DNN segmentation, and pixel-level analysis. PICoV was used to investigate the impact of DR on retinal perfusion heterogeneity. A significant correlation between PICoV and DR severity was observed, suggestive of a disruption to the perfusion autoregulation mechanisms in DR. This phenomenon was particularly pronounced temporal to the macula and aligns with existing literature suggesting that this area is particularly vulnerable to capillary drop out and other microvascular changes in DR. Our study is limited by a small sample size, a non-constant intra- and inter- imaging acquisition time, and external factors affecting pixel intensity.

## Data Availability

The datasets used and/or analysed during the current study are available from the corresponding author on reasonable request.

## Abbreviations

DR: Diabetic retinopathy
VEGF: Vascular endothelial growth factor
DM: Diabetes mellitus
OCTA: Optical coherence tomography angiography
VD: Vessel density
PD: Perfusion density
PICOV: Pixel intensity coefficient of variation
UBC: University of British Columbia
SVC: Superior vascular complex
DVC: Deep vascular complex
IPL: Inner plexiform layer
DNN: Deep neural network
ST: Superior temporal
SN: Superior nasal
IT: Inferior nasal
IN: Inferior nasal
FAZ: Foveal avascular zone
